# Supplementary material for: Are the healthcare providers willing and able to respond to disasters: An assessment of tertiary health care system of Khyber Pakhtunkhwa
Source: PLoS One. 2023 Nov 3;18(11):e0293720. doi: 10.1371/journal.pone.0293720 (PMC10624292; doi:10.1371/journal.pone.0293720)
Supplement: S1 File — (DOCX) [file pone.0293720.s001.docx]

**Anneture-1**

**Willingness & Ability Questionnaire**

For this research Willingness is defined as “voluntary intention to report to duty”

Ability is defined as “physical or mental health, personal obligations, other obligations, competence or circumstances that might hamper the ability to report to duty in his or her usual capacity”

I have been told about the research study and its purposes. I understand that my answers will be kept confidential and my identity will not be revealed to anybody. I hereby consent to answer all the questions in this questionnaire, to the best of my knowledge and ability.

1. Name: ____________________
2. Age: ____________________
3. Gender ____________________
4. Designation: ____________________
5. Working Experience: ____________________
6. Your Spouse is a Health Care Provider: Yes___ No___
7. Is your spouse suppose to work during disaster: Yes___ No___
8. Are you willing to do additional shifts if required: Yes___ No___
9. Preferred additional working shift: Morning__ Evening__ Night__ Any__
10. Pre existing Medical Conditions: Yes___ No___
11. Do you have Childcare Obligation: Yes___ No___
12. Do you have Elderly care Obligation: Yes___ No___
13. Level of Concern for Environmental Disaster: Mild/Low__ Moderate/Medium__ Severe/High__
14. Level of Concern for Earthquake : Mild/Low__ Moderate/Medium__ Severe/High__
15. Level of Concern for Treatable Infection: Mild/Low__ Moderate/Medium__ Severe/High__
16. Level of Concern for Untreatable Infection: Mild/Low__ Moderate/Medium__ Severe/High__
17. Level of Concern for Mass Causality Incidents: Mild/Low__ Moderate/Medium__ Severe/High__
18. Level of Concern for Chemical Spillage: Mild/Low__ Moderate/Medium__ Severe/High__
19. Level of Concern for Conventional War: Mild/Low__ Moderate/Medium__ Severe/High__
20. Level of Concern for Nuclear War: Mild/Low__ Moderate/Medium__ Severe/High__
21. Are you willing to respond to Environmental Disaster: Yes___ No___
22. Are you willing to respond to Earthquake: Yes___ No___
23. Are you willing to respond to Treatable Infection outbreak: Yes___ No___
24. Are you willing to respond to Untreatable infection outbreak: Yes___ No___
25. Are you willing to respond to Mass Causality Incidents: Yes___ No___
26. Are you willing to respond to Chemical Spillage: Yes___ No___
27. Are you willing to respond to Conventional War: Yes___ No___
28. Are you willing to respond to Nuclear War: Yes___ No___
29. Reason for unwillingness to respond to disaster: ___________________
30. Are you able to respond to Environmental Disaster: Yes___ No___
31. Are you able to respond to Earthquake: Yes___ No___
32. Are you able to respond to Treatable Infection outbreak: Yes___ No___
33. Are you able to respond to Untreatable infection outbreak: Yes___ No___
34. Are you able to respond to Mass Causality Incidents: Yes___ No___
35. Are you able to respond to Chemical Spillage: Yes___ No___
36. Are you able to respond to Conventional War: Yes___ No___
37. Are you able to respond to Nuclear War: Yes___ No___
38. Reason for not being able to respond to disaster: ____________________
