# Supplementary material for: Are the healthcare providers willing and able to respond to disasters: An assessment of tertiary health care system of Khyber Pakhtunkhwa
Source: PLoS One. 2023 Nov 3;18(11):e0293720. doi: 10.1371/journal.pone.0293720 (PMC10624292; doi:10.1371/journal.pone.0293720)
Supplement: Attachment — Submitted filename: Response To Reviewers.docx [file pone.0293720.s002.docx]

Response To Reviewers

# Journal Requirement

## Comment 1:

***In your Data Availability statement, you have not specified where the minimal data set underlying the results described in your manuscript can be found***

## Reply:

The data is now available at following URL on OSF

<https://osf.io/numeh/?show=revision&view_only=18026d1679104d709462a3ab4f54b6ed>

## Comment 2:

***Please include your full ethics statement in the ‘Methods’ section of your manuscript file. In your statement, please include the full name of the IRB or ethics committee who approved or waived your study, as well as whether or not you obtained informed written or verbal consent.***

## Reply:

Details added in the methodology section please see line 116-120. Regarding consent, details are given in the line 122-123

# Reviewer Comments

## Reviewer #1:

***You need to explain in the Methods section the statistical analysis and the statistical tests you have made and also because you are using a sample you will need to include in the Results section the confidence intervals or the parameters***

## Reply:

Statistical analysis and tests have now been described at the end of the methodology section. Please refer to line 125-128

All the results and tables have now been updated and 95% confidence intervals have been added.

Reviewer #2: ***Thank you for the opportunity to review this manuscript. The considerable work that has gone into the preparation and interesting findings reported are appreciated but the manuscript as a whole lacks sufficient organization and cohesive explication of major findings and implications.***

## Reply:

I apologies but I have tried to organize it to best of my ability. Can the reviewer please comment on section which needs improvement? And any suggestion will be highly appreciated

# Specific comments:

Introduction- ***the narrative is not well-focused, description of Khyber Pakhtunkhwa (KP) province cursory, local demographics are not described***

## Reply:

Information related to KP health system has been added in addition to information related to disasters and affects that were already mentioned. Please refer to lines 47-59

Methodology- ***while using a prior tool that has been adapted with additional scenarios, this survey does not differentiate or provide further details about what specific contexts within the scenario willingness to respond applies***

## Reply:

This survey builds upon the prior tool and added additional disaster scenarios which have not been studied by the previous researches. The additional disaster scenarios are very relevant to the KP province and Pakistan and this survey has assessed the willingness and ability of HCPs for these scenarios. Furthermore this study has also explored the proportion of HCPs who are both willing and able to respond to different disaster scenarios

Discussion- ***reasonable discussion of findings, but implications of findings not addressed; no limitations of study described.***

## Reply:

Implications have been discussed in the last couple of paragraphs of Conclusion (please refer to lines 421-432)

Limitation section added please refer to lines 433-443

Tables- ***some typos "mass causality***

## Reply:

Manuscript was reviewed and spelling and grammatical mistakes were corrected

Editor Comments 20^th^ April

Comment 1

Thank you for including your ethics statement on the online submission form:

"Ethical approval was obtained from Ethical Review Board of Khyber Medical College. Data Collection permissions were obtained from hospital/medical directors of the respective hospitals

The healthcare providers were given questionnaire with information about the study and consent statement. The healthcare providers were asked to return the filled questionnaire and by doing so consent were implied."

To help ensure that the wording of your manuscript is suitable for publication, would you please also add this statement at the beginning of the Methods section of your manuscript file.

Reply

Added at the beginning of the methodology section

Comment 2

Thank you for stating the following:

"Yes - all data are fully available without restriction;

All relevant data are within the manuscript and its Supporting Information files.

This work is part of my PhD thesis. As per the directions of the institute, we are not suppose to make publicly available our data set. The reason behind is that it may be used by someone else in their publication and when our thesis plagiarism is checked, it may appear that a copied data is used in the thesis

The data is available with author and if the journal wishes to have the data, SPSS file can be sent to journal for verification of results written in the paper. Additionally all the tables from which results are extracted are already mentioned in the article"

Please know that in the interest of long-term data availability, PLOS data policy states that it is not acceptable for an author to be the sole named individual responsible for ensuring data access

Reply

The data is now available at following URL on OSF

<https://osf.io/numeh/?show=revision&view_only=18026d1679104d709462a3ab4f54b6ed>
