# Supplementary material for: Are the healthcare providers willing and able to respond to disasters: An assessment of tertiary health care system of Khyber Pakhtunkhwa
Source: PLoS One. 2023 Nov 3;18(11):e0293720. doi: 10.1371/journal.pone.0293720 (PMC10624292; doi:10.1371/journal.pone.0293720)
Supplement: Attachment — Submitted filename: Response to Reviewer .docx [file pone.0293720.s004.docx]

# Response to Reviewers

## Editor Comments

**Add the ethical approval number in the methodology section**.

Reply: Mentioned and highlighted

**Add the name of the software used for statistics and more details regarding the analysis. Chi-square test is not used to measure association, please mention why did you used Cramer V statistical tests specifically.**

Reply:	Software name now mentioned in methodology section

Cramer V was used to assess strength of association

**Why the authors did not do some regression analysis. The data could be presented better than in the format of proportion only. You could have done some score scale for each outcome of interest and then stratify participants demographic based on this score, then you can do regression analysis to identify predictors. Refer to this article as a reference to understand what i mean (**[**https://pubmed.ncbi.nlm.nih.gov/32578943/**](https://pubmed.ncbi.nlm.nih.gov/32578943/)**)**

Reply: Analysis was made as advised by the PhD supervisor

**The manuscript required professional English language editing to enhance the flow. Please provide us with certificate of accomplishing this upon re-submission.**

Reply: The manuscript has already been edited by the university editor. The university editors don’t issue certificate. If required email copy can be provided

**Add further details about sample size calculation including sample size of your population and what is the minimum required sample size (include the formula used).**

Reply: Details added

**-Describe exactly how did you apply your recruitment procedure (stratified random sampling).**

Reply : Details added

**Further details are needed regarding questionnaire tool and their assessment scale (format of questions).**

Reply: Tool has been uploaded as a separate file

## Reviewer #3:

This is a review of the paper entitled "Are the healthcare providers willing and able to respond to disasters: An assessment of tertiary health care system of Khyber Pakhtunkhwa." Thank you for the opportunity to review this manuscript. Unfortunately, I still believe the manuscript lacks sufficient organization and cohesive explication of major findings and implications.

I understand that there has been a lot of work and effort spent on the data collection and analysis since it is based on a dissertation, but it’s not logical to fit all your findings in one academic paper. The current manuscript goes back and forth between such big topics and does not really dig deep into one of them. The paper still lacks focus and does not have a clear message. I don’t need 10 pages of tables and data to know that not everyone in the medical force will be not willing or not able to respond; we already know that. I need to see what this article has to add!

It would be more beneficial to see some more in-depth discussion and clarifications; hopefully, the points raised here will aid the process:

**1) Submit a blinded manuscript (no names) next time, because this peer review is a blind process. There is no need for me to see the names of the authors and affiliations in the submittal.**

Reply: Names Removed

**2) There is no need to submit the original report again; we already have it in the system.**

Reply: Only revised version is shared

**3) Don’t use the track changes option in Microsoft Words in the modified manuscript; it makes the document looks messy. Simply highlight only the major changes!**

Reply: Corrected as suggested

**4) Please do not use the 'natural disasters' misnomer! The word ‘Natural’ in a disaster context, suggests the naturalization and normalization of disasters, which is a misleading idea! So much evidence exists pointing out that disasters are not natural; this argument is not new (see O'Keefe et al, 1976: "Taking the 'Naturalness' out of 'Natural Disaster'', as well as more recent work by Kelman or Chmutina et al., in addition to a popular #nonaturaldisasters campaign on Twitter). Please use the term ‘Natural Hazards’ instead, or use ‘disaster’ without the word ‘natural’**.

Reply: Corrected and highlighted

**5) In the methodology, I need to know more information about the actual population and how the sample size was determined, and what was the confidence level and confidence interval for the sample. I also want to see the demographics of the original population which the sample was taken from.**

Reply: Mentioned and highlighted

**6) Your study is based on (Qureshi, K., Gershon, R. R., Sherman, M. F., Straub, T., Gebbie, E., McCollum, M., Erwin, M. J., & Morse, S. S. (2005). Health care workers’ ability and willingness to report to duty during catastrophic disasters. Journal of Urban Health, 82(3), 378–388.) Try to organize your tables and the data you collected in the same way they did! Also, add the limitation in their study to your paper as it should apply here as well. In future studies, try to use a rising scale for your survey, and give the participants more options, for example (not able, able, not sure) 3-point scales are not a good tool to use. Use 5 or 7 points scale**s.

Reply: Limitations are added as suggested

**7) You reflected on the validity at mutable locations in the paper, but you didn’t mention anything about reliability/consistency measures. I understand that the survey tools are previously validated and commonly used, but even for those, measuring reliability/consistency can be helpful. If you have not measured reliability/consistency for your data collected, can you please reflect on that in the limitation as well?**

Reply: Mentioned in limitations

**8) In your result section, you don’t need to repeat findings that I can see in the tables in the texts as well.**

Reply: Results are trimmed and findings which were shown in the table were removed from narration

**9) You have too many tables! Reorganize them and try to merge them somehow to make their presentations better. You don’t need to report all the data you collected! If there is no significant finding in one of the tables, don’t include it.**

Reply: 2 tables removed

**10) Reorganize your discussion and make it in multiple subsections.**

Reply Discussion has been trimmed with two clear sub sections. One addressing willingness and other ability

**11) You need a recommendation section. This is very important! I need to see how I can use your findings! Add the recommendation as a substation at the end of the discussion. Also, the paper does not discuss the social component of the issue or impact on the society in general or what needs to be done to help fix the problem. Integrating mental health in disaster response plans is a very critical topic these days and there are a lot of papers published about it. Adding that to the paper is very important, you can add it to the discussion section, or even make a separate recommendation section. You can start here:
• Cohen, S., & Abukhalaf, A. H. I. (2021). Necessity to Plan and Implement Mental Health Disaster Preparedness and Intervention Plans. Academia Letters. https://doi.org/10.20935/AL3507
• Cohen, S., Abukhalaf, A.H.I. (2021). COVID-19’s Negative Mental Health Impact Goes Well Beyond Standard At-Risk Populations: Action Needs To Be Taken to Combat Long-term Nationwide Emotional Disruption. Academia Letters, Article 3621. https://doi.org/10.20935/AL3621
• Cohen, S., von Meding, D., Abukhalaf, A.H.I. (2021). Successful Pandemic and Disaster Mental Health Preparedness Requires Widespread Community Collaboration. Academia Letters, Article 3987.** [**https://doi.org/10.20935/AL3987**](https://doi.org/10.20935/AL3987)

Reply: Added

**12) The limitation section should be much bigger based on this study and the methodology chosen. Think about the limitations in your study again and expand that section. Move the limitation section to before the conclusion.**

Reply: Additions done in limitations section

**13) Add an appendix with the survey question at the end of the report. If the survey was conducted in a different language, provide a translated version of the survey in the English language.**

Reply: Survey Tool uploaded as a separate document

**14) If there are no Acknowledgments, remove that section instead of writing (not applicable).**

Reply: Removed

I understand that these comments may seem like a lot to you, but it’s better for you to work on fixing the manuscript and to keep those comments in mind as a good practice for future submittals. Your paper should be well-written, well-organized, well-presented, with a very specific focus and direct and clear massage of implications.

## Reviewer #4:

The significant effort put into the preparation and the intriguing discoveries presented are great, but the paper has some minor issues and lacks some of the key findings and consequences.
The discussion is an important part of the paper. It should have a reasonable discussion about the results and findings. However, the most important part is the comparison and relating the findings to the similar studies and alternative description of the study. It should be followed by the implication of the study and its solutions to make the study stronger and more reasonable. Finally, the suggestion, which can provide the opportunity for future studies, is worth adding to this part.

**1. In the introduction part, there are some statistics about the healthcare facilities in Khyber Pakhtunkhwa, lines 47 to 55, that do not have references. Also, the description of the location and province are not described clearly enough.**

Reply: Reference added

**2. In methodology, Line 94, explains the perception method based on the quantitative method in the methodology section. Also, describe the statistical tests that you have done for the analysis.**

Reply: Statistical test have been mentioned in methodology

**3. Discussion part covers the discussion of results in detail, but there is not a strong implication, comparison, and suggestions.**

Reply: Recommendations have been added. Comparison with other studies already discussed in the discussion section

**4. Figure 1 is not clear, create a clear figure with better resolution.
Table 11, is not complete. It has messed.**

Reply: The word was converted to .tiff format and resolution remains the same. I tried and got same results. Table 11 is now table 9 and its complete
