# Supplementary material for: Are the healthcare providers willing and able to respond to disasters: An assessment of tertiary health care system of Khyber Pakhtunkhwa
Source: PLoS One. 2023 Nov 3;18(11):e0293720. doi: 10.1371/journal.pone.0293720 (PMC10624292; doi:10.1371/journal.pone.0293720)
Supplement: Attachment — Submitted filename: Reviewer Comments.docx [file pone.0293720.s003.docx]

**Reviewer Comments**

This is a review of the paper entitled "Are the healthcare providers willing and able to respond to disasters: An assessment of tertiary health care system of Khyber Pakhtunkhwa." Thank you for the opportunity to review this manuscript. Unfortunately, I still believe the manuscript lacks sufficient organization and cohesive explication of major findings and implications.

I understand that there has been a lot of work and effort spent on the data collection and analysis since it is based on a dissertation, but it’s not logical to fit all your findings in one academic paper. The current manuscript goes back and forth between such big topics and does not really dig deep into one of them. The paper still lacks focus and does not have a clear message. I don’t need 10 pages of tables and data to know that not everyone in the medical force will be not willing or not able to respond; we already know that. I need to see what this article has to add!

It would be more beneficial to see some more in-depth discussion and clarifications; hopefully, the points raised here will aid the process:

1) Submit a blinded manuscript (no names) next time, because this peer review is a blind process. There is no need for me to see the names of the authors and affiliations in the submittal.
2) There is no need to submit the original report again; we already have it in the system.
3) Don’t use the track changes option in Microsoft Words in the modified manuscript; it makes the document looks messy. Simply highlight only the major changes!
4) Please do not use the 'natural disasters' misnomer! The word ‘Natural’ in a disaster context, suggests the naturalization and normalization of disasters, which is a misleading idea! So much evidence exists pointing out that disasters are not natural; this argument is not new (see O'Keefe et al, 1976: "Taking the 'Naturalness' out of 'Natural Disaster'', as well as more recent work by Kelman or Chmutina et al., in addition to a popular #nonaturaldisasters campaign on Twitter). Please use the term ‘Natural Hazards’ instead, or use ‘disaster’ without the word ‘natural’.
5) In the methodology, I need to know more information about the actual population and how the sample size was determined, and what was the confidence level and confidence interval for the sample. I also want to see the demographics of the original population which the sample was taken from.
6) Your study is based on (Qureshi, K., Gershon, R. R., Sherman, M. F., Straub, T., Gebbie, E., McCollum, M., Erwin, M. J., & Morse, S. S. (2005). Health care workers’ ability and willingness to report to duty during catastrophic disasters. Journal of Urban Health, 82(3), 378–388.) Try to organize your tables and the data you collected in the same way they did! Also, add the limitation in their study to your paper as it should apply here as well. In future studies, try to use a rising scale for your survey, and give the participants more options, for example (not able, able, not sure) 3-point scales are not a good tool to use. Use 5 or 7 points scales.
7) You reflected on the validity at mutable locations in the paper, but you didn’t mention anything about reliability/consistency measures. I understand that the survey tools are previously validated and commonly used, but even for those, measuring reliability/consistency can be helpful. If you have not measured reliability/consistency for your data collected, can you please reflect on that in the limitation as well?
8) In your result section, you don’t need to repeat findings that I can see in the tables in the texts as well.
9) You have too many tables! Reorganize them and try to merge them somehow to make their presentations better. You don’t need to report all the data you collected! If there is no significant finding in one of the tables, don’t include it.
10) Reorganize your discussion and make it in multiple subsections.
11) You need a recommendation section. This is very important! I need to see how I can use your findings! Add the recommendation as a substation at the end of the discussion. Also, the paper does not discuss the social component of the issue or impact on the society in general or what needs to be done to help fix the problem. Integrating mental health in disaster response plans is a very critical topic these days and there are a lot of papers published about it. Adding that to the paper is very important, you can add it to the discussion section, or even make a separate recommendation section. You can start here:

- Cohen, S., & Abukhalaf, A. H. I. (2021). Necessity to Plan and Implement Mental Health Disaster Preparedness and Intervention Plans. Academia Letters. <https://doi.org/10.20935/AL3507>
- Cohen, S., Abukhalaf, A.H.I. (2021). COVID-19’s Negative Mental Health Impact Goes Well Beyond Standard At-Risk Populations: Action Needs To Be Taken to Combat Long-term Nationwide Emotional Disruption. Academia Letters, Article 3621. <https://doi.org/10.20935/AL3621>
- Cohen, S., von Meding, D., Abukhalaf, A.H.I. (2021). Successful Pandemic and Disaster Mental Health Preparedness Requires Widespread Community Collaboration. Academia Letters, Article 3987. <https://doi.org/10.20935/AL3987>

12) The limitation section should be much bigger based on this study and the methodology chosen. Think about the limitations in your study again and expand that section. Move the limitation section to before the conclusion.
13) Add an appendix with the survey question at the end of the report. If the survey was conducted in a different language, provide a translated version of the survey in the English language.
14) If there are no Acknowledgments, remove that section instead of writing (not applicable).

I understand that these comments may seem like a lot to you, but it’s better for you to work on fixing the manuscript and to keep those comments in mind as a good practice for future submittals. Your paper should be well-written, well-organized, well-presented, with a very specific focus and direct and clear massage of implications.
